# Bibliography

**Published:** 1916-12

**Authors:** 


### BIBLIOGRAPHY

WHO'S WHO IN DENTISTRY. This is a small book of 230 pages, gives descriptive information about some 200 more or less prominent members of the dental profession in the United States and Canada. It is edited by Dr. Samuel Greif, of Brooklyn, N. Y., and is published by The Who's Who Dental Publishing Co., of New York City. The book is well compiled, and will prove very useful as a place to get important data as to the lives of men who are active in professional work. It is a very handy and useful book and should be in the library of every dentist who reads the current literature, that he may get into sympathetic relations with the men whose ideas he reads about. It may be had from dental depots.

ORAL ABSCESSES. This is a book of 200 pages written by Dr. Kurt H. Thoma, lecturer on Oral Histology and Pathology in Harvard University Dental School, and instructor in Dental Anotomy in Harvard Medical School. Dr. Thoma is the author of several other books on dental subjects, and is well known for his contributions to dental current literature. The book aims to state in well chosen and explicit text the nature and results of infectious diseases associated with the teeth. The text is strongly supplemented with beautifully colored drawings and many illustrations which add materially to the value of the book. Schematic drawings, photographic illustrations, and radiographs help to make the subject clear and comprehensible. It is rarely that a dental book has appeared that was so well illustrated. It is a timely book, as the subject of infection of the general system is now so actively under observation by both the dental and medical professions, especially as it is related to diseased teeth. The classification of the material is systematic and sequential. Much of the value of the work we are inclined to believe will be its treatment from both the scientific and technical aspects. As only one distinct field of pathology is discussed, and that by a man who has studied it both scientifically and technically from the viewpoint of the practical dentist, we predict that it will become a standard text and reference book for students and practitioners as well.

The publishers, Ritter and Co., of Boston, deserve great credit for the artistic typing and illustrating. It is a beautifully made book, and because of the rare combination of worth and style it will appeal most strongly to lovers of good books.

